# Survey on association between Mycoplasma hominis endocervical infection and spontaneous abortion using Polymerase Chain Reaction

**Published:** 2016-03

**Authors:** Fariba Farhadifar, Mazaher Khodabandehloo, Rashid Ramazanzadeh, Samaneh Rouhi, Amjad Ahmadi, Ebrahim Ghaderi, Daem Roshani, Nasrin Soofizadeh, Masoomeh Rezzaii

**Affiliations:** 1 *Social Determinants of Health Research Center, Kurdistan University of Medical Sciences, Sanandaj, Iran.*; 2 *Department of Genecology, Kurdistan University of Medical Sciences, Sanandaj, Iran.*; 3 *Cellular and Molecular Research Center, Kurdistan University of Medical Sciences, Sanandaj, Iran.*; 4 *Microbiology Department, Kurdistan University of Medical Sciences, Sanandaj, Iran.*; 5 *Student Research Committee, Kurdistan University of Medical Sciences, Sanandaj, Iran.*

**Keywords:** *Mycoplasma hominis*, *Spontaneous Abortion*, *Miscarriage*, *Women*.

## Abstract

**Background:**

Mycoplasma infections are suggested as etiology of adverse pregnancy outcomes.

**Objective:**

The aim of this study was to evaluate the association of Mycoplasma hominis (M. hominis) infection and spontaneous abortion among pregnant women.

**Materials and Methods:**

In this case-control study that was conducted from August 2012 to January 2013, totally, 109 women were included with spontaneous abortion with gestational ages of 10-20 weeks (Cases), and 109 women with normal pregnancy with gestational ages between 20-37 weeks (Controls) in Sanandaj, Iran. Using specific primers and extracted DNA from endocervical swabs, a PCR test was conducted for detection of M. hominis infection in women. For comparison of qualitative and quantitative variables, independent Fisher tests were used and p<0.05 was considered significant.

**Results:**

The total frequency of M. hominis infection was 6 (2.75%) in women. The frequency of M. hominis infection was 2 (1.83%) in the case group (spontaneous abortion) and 4 (3.66%) in the control group, respectively. In both case and control groups, no association was seen between M.hominis infection and spontaneous abortion (OR=0. 49, CI 95%: 0.08-2.73, p=0. 683).

**Conclusion:**

M. hominis was positive in the genital tract of some pregnant women, but it was not associated with spontaneous abortion. However, to prevent adverse pregnancy outcomes in women, foetus and neonate, routine screening and treatment for the genital Mycoplasma is recommended.

## Introduction

Adverse pregnancy outcomes are abortion (miscarriage), stillbirth and preterm labour (PTL). Abortion is defined as spontaneous termination of pregnancy before 20 wk and stillbirth happens after 20 wk of gestation, but before birth. PTL occurs before 37 wk of gestation, due to uterine contractions and shortening of cervix. All of these outcomes could be caused by genitourinary and intrauterine Mycoplasma infections (1-3). 

Mycoplasma hominis (M. hominis) and Ureaplasmaspp (U. parvum and U. urealyticum) may exist in pregnant and non- pregnant genital tract of women. M. hominis is specifically associated with endometritis and preterm birth (PTB) (4). Clinical and experimental studies suggest that Mycoplasma cervicovaginal colonization and amniotic fluid infection induce an inflammatory response resulting in chorioamnionitis, PTL or preterm premature rupture of the membranes (PPROM) (1). M. hominis was isolated from amniotic fluid in 30% of women with intra-amniotic infections and proved to be associated with PTB (5). M. hominis and U. urealyticum were detected in vaginal cultures, and patients carrying both organisms simultaneously had more severe adverse pregnancy outcomes compared to patients in PTB or PPROM who were only positive for U. urealyticum (6). 

Here are some studies that have shown Mycoplasma infection in women. Kwak *et al* in Korea using vaginal cultures for M. hominis and U. urealyticum in women with PPROM, showed that prevalence of positive vaginal fluid cultures for genital Mycoplasma was 62.5% and being infected by these organisms will cause a decrease in gestational age at birth, birth weight, and significant increases in incidences of PTB, and chorioamnionitis (6). 

Choi *et al* in Korea, using PCR and culture method, reported that in 126 pregnant women vaginal swab specimens, U. urealyticum and M. hominis were detected in 62.7% and 12.7% of cases, respectively (7). Perni *et al* in USA using PCR coupled with enzyme-linked immunosorbent assay (ELISA) reported that in 179 amniotic fluids of pregnant women, M. hominis was present in 6.1% of amniotic fluids. In their study, 5 women had spontaneous preterm birth with intact membranes and were positive for either U. urealyticum or M. hominis (8). In Iran, there is no established national program for prenatal screening of women for Mycoplasma infections (9). 

The aim of this case-control study was to evaluate the frequency of M. hominis infections using PCR method among two groups of pregnant women (spontaneous abortion and normal pregnancy) and its association with spontaneous abortion.

## Materials and methods

**Subjects**

This case-control study was conducted from August 2012 to January 2013. Pregnant women who admitted to midwifery practices in obstetrics, gynecology wards and prenatal clinic of Beasat Hospital located in Sanandaj, Iran, were included in this study. This study had the written consent of all its participants in both groups; case and control and was approved by the Ethics Committee of the Kurdistan University of Medical Sciences.

109 women were included (aged 19-43 years) with spontaneous abortion with gestational ages between 10-20 weeks (Cases), and 109 (aged 19-43 years) women with normal pregnancy with gestational ages between 20-37 weeks (Controls). Women in each group, cases and controls, were divided into two parts; women ˃25 and women ˂25 years of age. 

These included 26 women over 25 years of age and 83 women below 25 years of age in cases and also 26 women over 25 years of age and 83 women below 25 years of age in controls, randomly. Demographic data such as age, place of residence, education, occupation and obstetrical and medical data such as number of childbirth, gestational age, history of miscarriage, premature delivery, genital infection, urinary infection, alcohol consuming, smoking before and during pregnancy, contraceptive usage before pregnancy and urinary tract infection (UTI) of their husbands were questioned by gynecologist. 

In addition, in order to determine the first day of their last menstrual, ultrasound scan tests were performed for better estimation of their gestational age. To eliminate the threat of chromosomal abnormalities and probability of genetically miscarriage, fetal health assessment tests were done between 11-13 wk of gestation, including nuchal translucency (NT), double tests such as Pregnancy Associated Plasma Protein A (PAPPA) and free β human chorionic gonadotropin (HCG). Also, at 15-17 wk of gestation, the confirmatory triple tests (αfetoprotein, titer of βHCG and unconjugated estradiol) were performed for elimination of probable neural tube and chromosomal anomalies. 

Inclusion criteria were pregnancy, having sexual activity, gestational age and negative double and triple tests. Exclusion criteria were as follows: the use of antibiotics two weeks before sampling, immunodeficiency, chronic diseases (diabetes, endocrine disorders, and hypertension), vaginal infections and recurrent miscarriage due to anatomic complications. The groups were matched by age, gestational age, and numbers of pregnancies. Endocervical swab specimens were taken from all women before any pregnancy outcomes. Samples were immediately taken into sterile tubes containing 5 ml of PBS (phosphate buffered saline) in 15 ml Falcon tubes and placed at -70^o^C until DNA extraction.

**DNA extraction**

Tubes containing cervical swab specimens were centrifuged at 6000 rpm for 30 min. Then, supernatant was discarded and sediment was poured into 1.5 ml microtube. Then sediment was used for DNA extraction using DNA extraction kit (High pure PCR Template Preparation, Roche, Germany). To avoid DNA being broken, DNA sample was aliquoted into 0.2 ml microtubes and maintained at -70^o^C until conducting PCR test.

**PCR test for M.hominis detection**

We designed specific primers for 16s ribosomal gene of M.hominis genome deposited in GeneBank. The primer sequences were as: Forward: 5'-CAA TGG CTA ATG CCG GAT ACG C-3' and Reverse: 5'-GGT ACC GTC AGT CTG CAA T-3'. Using designed primers and extracted DNA, a PCR test was conducted. Total volume of PCR reaction was 25 µl using premade PCR master mix (CinnaGen, Iran). The length of M.hominis PCR product was 334 bp.

The PCR amplification program was performed in Termocycler (Eppendorf, Germany): Initial denaturation at 94^o^C for 10 min, followed by 30 cycles of denaturation at 94^o^C for 1 min, annealing at 64^o^C for 30 sec, extension at 72^o^C for 1 min, and final extension at 72^o^C for 10 min. PCR products were separated by Gel electrophoresis apparatus (GE-100, China) in 1.5% gel agarose, stained with ethidium bromide, visualized by UV light and photographed. The PCR positive control was DNA extracted from M. hominis.

**Statistical analysis**

The data were entered into the SPSS statistic software (Statistical Package for the Social Sciences, version 16.0, SPSS Inc, Chicago, Illinois, USA) and analyzed. For comparison of qualitative variables, independent Fisher test was used, and p<0.05 was considered significant.

## Results

**Figure 1:**
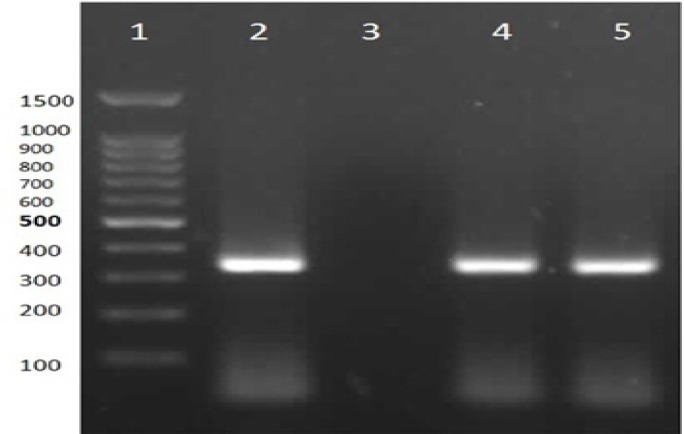
PCR test for *M.hominis* detection: Lane 1) 100 bp DNA Ladder (CinnaClon, Iran), lane 2) PCR positive control, Lane 3) negative control, Lanes 4 and 5) positive PCR products (334 bp) (resolution of image is **300 dpi**

The total frequency of M. hominis infection in two groups of women (normal pregnancy and spontaneous abortion) was 6 (2.75%). The frequency of M. hominis infection was 2 (1.83%) in case (spontaneous abortion) and 4 (3.66%) in control group. The frequencies of M. hominis infection among <25 years old women and >25 years old women in case and control groups are shown in table I. All women included in study were living in urban areas. None of women had reported a history of miscarriage in their previous pregnancies. Also, representative stained agarose gel electrophoresis following the PCR assay is shown in figure 1. 

The age of subjects ranged from 19-43 years old (29.6±5.9) in case and 19-42 years old in control group (27.8±4.87) (n=109); median age in both groups was 25. The ranges of gestational age in case and control groups were 10-20 wk (15±1) and 20-30 wk (28±1), respectively. The Fisher test showed that there was no significant association between M. hominis endocervical infection and spontaneous abortion (OR=0.49, CI 95%: 0.08-2.73, p=0.05).

**Table I:** Demographic data, risk factors and prevalence of M.hominis infections in women with spontaneous abortion (Case group) and women with normal delivery (Control group

**Variables**		**Spontaneous abortion**	**Normal delivery**	**p-value**
Age (Year of birth)*		(29.6 ±**5.9)**	(27.8 **± 4.87)**	
M. hominis in women a^,^ **				
	<25 years old	0 (0%)	2 (1.83%)	0.83
	≥25 years old	2 (1.83%)	2 (1.83%)	
Education				
	Illiterate	5 (4.58%)	3 (2.75%)	0.08
	Primary	49 (44.95%)	33 (30.27%)	
	High school	36 (33.02%)	44 (40.36%)	
	Academic	19 (17.43%)	29 (26.60%)	
Occupation				
	Housekeeper	97 (88.99%)	97 (88.99%)	1
	Employee	12 (11.00%)	12 (11.00%)	
History of smoking		0 (0%)	3 (2.75%)	0.43
History of preterm premature rupture of the membranes		5 (4.58%)	1 (0.91%)	0.084
History of Vaginal infection		11 (10.09%)	5 (4.58%)	0.115
History of Urinary infection		9 (8.25%)	8 (7.33%)	0.801
Prevalence of M. hominis infection		2 (1.83%)	4 (3.66%)	0.68

* Data are presented as mean±SD. (n=109)

** Data are presented as n (%).

a , Women who were infected by M. hominis bacteria.

## Discussion

Mycoplasmas are associated with different patterns of infection in pregnant mothers and their infants. According to different studies, M.hominis infection may be associated with adverse pregnancy outcomes. Studies have shown that 40-80% of women are colonized with genital Mycoplasmas (1, 10). In present study, total frequency of M.hominis infection in women groups (normal pregnancy and spontaneous abortion) was 6 (2.75%). Bayraktar *et al* using Mycoplasma IST-2 kit and A7 agar medium on 1000 pregnant women found that 12 symptomatic pregnant women had spontaneous abortion and from those, 12 (66.66%) had been colonized with M.hominis and/or U.urealyticum (11). 

In another study on endocervical swab samples using PCR, Govender *et al* reported a co-colonization of M.hominis and U.urealyticum in women aged ≥21 years. In their study, there was no association between colonization of M. hominis, U.urealyticum, U.parvum and labour outcome (12). Najar Peerayeh and Samimi in study on 312 endocervical swab samples that were taken from infertile women using culture and PCR reported that genital Mycoplasmas were detected in 35.5% of samples of both culture and PCR methods. In this study, sensitivities of 91.8% and 53% were found for PCR and culture methods, respectively (13).

Although, culture is the gold standard test for identification of M. hominis, but colonial identifications performed using only human eye and a dissecting microscope, may cause for misidentification, false-positive results and also false-negative results (14). But for detecting these bacteria in clinical samples, the PCR assay helps to have more confident results about their incidence and distribution. In recent years, PCR has become a readily available and reliable method for detecting mollicutes in human genital tract (15). This method is more sensitive, less time consuming and is able to differentiate genera and species. The PCR assay has been used to detect M. hominis infections in women and has been proved to be more sensitive and specific than culture method (16). 

Also, the detection rate of M. hominis by PCR was higher than culture method (7). So, difference in analytical methods used in studies may cause disagreement among researchers’ results (17). Baczynska *et al* using quantitative PCR (qPCR) showed that 2.4% of cervical samples of women who had attended to fertility clinics were infected with M.hominis and Rodrigues *et al* using PCR in another study that was performed on cervical swabs of 224 women, reported that M.hominis was found in 21.9% of samples (18, 19). The frequency of M.hominis infection in our study was 2 (1.83%) in case (spontaneous abortion) and 4 (3.66%) in control group. Results in our research are close to Baczynska’s study, but rate of infection in Rodrigues’s study is higher than ours. The different rates of bacterial infection in women may be linked to development of disease, the more the disease advances, the more will be infected (18). 

In present study, there were no significant associations between M.hominis infection and spontaneous abortion in women. The lack of association may be explained by population differences. In addition, we did not consider co-infections with other microorganisms in this study. Co-infections with other microorganisms may act as confounding factors. Also, there is still no consensus regarding role of detected M.hominis in adverse pregnancy outcomes. In some previous studies that culture or PCR method was used for M.hominis infection, no significant association between M.hominis infection and adverse pregnancy outcomes was detected (6, 8, 17, 18). However, other studies have shown that the presence of M.hominis is correlated with development of pelvic inflammation, spontaneous abortions and infertility (12). Studies that were based on amniotic fluid samples (using culture or/and PCR methods) have shown a significant association between M.hominis and adverse pregnancy outcomes (21, 22). 

M.hominis affects host’s immune response and it is proved that inflammatory cytokines play a critical role in regulating response to M.hominis infections (23). Immune response and its tropism for different pathogens affect pregnant women and their susceptibility to severity of infectious diseases (24). In Aydin *et al* study, PCR method was performed on 96 pregnant women that had delivered by caesarean section and 124 non-pregnant women for cervical C.trachomatis, U.urealyticum and M.hominis infections. The results showed that the prevalence of C.trachomatis and U.urealyticum infections higher than M.hominis (25). 

In Baczynska *et al* study, lower genital tract carriage rate of M.genitalium was determined using Real-Time PCR and the results were compared to the carriage rates of M.hominis and C. trachomatis among 102 women requesting termination of pregnancy. M.genitalium was detected in one swab sample (0.98%) only, while the prevalence of C.trachomat was high (15.69%) and the rate of M.hominis colonization was 18.63%. Also, between the prevalence of M.hominis infection and age groups was a significant difference (26). Zdrodowska-Stefanow *et al* by using the Mycoplasma IST 2 kit on 541 women from gynaecological and STD out-patient clinics, which aged from 18 to 55 years, showed that the rates of U. urealyticum and M.hominis were 29.8% and 3.7%, respectively. 

Also, in Zdrodowska-Stefanow’s study, sexual Mycoplasmal infections were most frequently among STD clinic patients (their age ranged from 26 to 30 years) and it was associated with sexual activity (27). In present study, age range in control group was 19-42 years, with Mean±SD of 27.8±4.87; and in case group it was 19-43 years with Mean± SD of 29.6±5.9 years. These results indicated presence of infections in sexually active women. Also, history of vaginal infection in case group was somewhat higher than control group, but not statistically significant. 

Even though presence of these microorganisms in vaginal flora may be insufficient to cause problems, but combined with other factors such as bacterial vaginosis or cervical incompetence, they may induce spontaneous abortion. Another approach is whether antimicrobial therapy of M.hominis in pregnant women can reduce the adverse pregnancy outcomes. Prevalence of M.hominis is associated with many other factors, including race, socioeconomic status, and hormonal changes in pregnancy, and number of sexual partners, maternal and gestational ages, site of sampling, detection method used and co-infection with other microorganisms. 

On the other hand, these bacteria can be a part of normal genital flora and most of infections may be asymptomatic. Therefore, their role in adverse pregnancy outcomes is controversial (28, 29). Hence, in studying prevalence of this bacterium in women, all of these factors should be considered as they explain the variations in prevalence between different studies.

## Conclusion

The results of our study showed that M.hominis was positive in genital tract of some pregnant women, but it was not associated with spontaneous abortion. However, to prevent adverse pregnancy outcomes in women, foetus and neonate routine screening and treatment for the genital mycoplasmas is recommended. 

## Note

***This article extracted from M.Sc. thesis. (Amjad Ahmadi)***
